# Advances in Biodetoxification of Ochratoxin A-A Review of the Past Five Decades

**DOI:** 10.3389/fmicb.2018.01386

**Published:** 2018-06-26

**Authors:** Wenying Chen, Chen Li, Boyang Zhang, Zheng Zhou, Yingbin Shen, Xin Liao, Jieyeqi Yang, Yan Wang, Xiaohong Li, Yuzhe Li, Xiao L. Shen

**Affiliations:** ^1^School of Public Health, Zunyi Medical University, Zunyi, China; ^2^Experimental Teaching Demonstration Center for Preventive Medicine of Guizhou Province, Zunyi Medical University, Zunyi, China; ^3^Department of Pharmacology, Perelman School of Medicine, University of Pennsylvania, Philadelphia, PA, United States; ^4^Beijing Advanced Innovation Center for Food Nutrition and Human Health, College of Food Science and Nutritional Engineering, China Agricultural University, Beijing, China; ^5^Department of Food Science and Engineering, School of Science and Engineering, Jinan University, Guangzhou, China; ^6^Department of Food Quality and Safety, Institute of Food Science and Technology, Chinese Academy of Agricultural Sciences, Beijing, China; ^7^Department of Food and Bioengineering, Beijing Agricultural Vocational College, Beijing, China; ^8^China National Center for Food Safety Risk Assessment, Beijing, China

**Keywords:** Ochratoxin A, biodegradation, adsorption, bacteria, filamentous fungi, yeast

## Abstract

Ochratoxin A (OTA) is a toxic secondary fungal metabolite that widely takes place in various kinds of foodstuffs and feeds. Human beings and animals are inevitably threatened by OTA as a result. Therefore, it is necessary to adopt various measures to detoxify OTA-contaminated foods and feeds. Biological detoxification methods, with better safety, flavor, nutritional quality, organoleptic properties, availability, and cost-effectiveness, are more promising than physical and chemical detoxification methods. The state-of-the-art research advances of OTA biodetoxification by degradation, adsorption, or enzymes are reviewed in the present paper. Researchers have discovered a good deal of microorganisms that could degrade and/or adsorb OTA, including actinobacteria, bacteria, filamentous fungi, and yeast. The degradation of OTA to non-toxic or less toxic OTα via the hydrolysis of the amide bond is the most important OTA biodegradation mechanism. The most important influence factor of OTA adsorption capacity of microorganisms is cell wall components. A large number of microorganisms with good OTA degradation and/or adsorption ability, as well as some OTA degradation enzymes isolated or cloned from microorganisms and animal pancreas, have great application prospects in food and feed industries.

## Introduction

**Figure 1:**
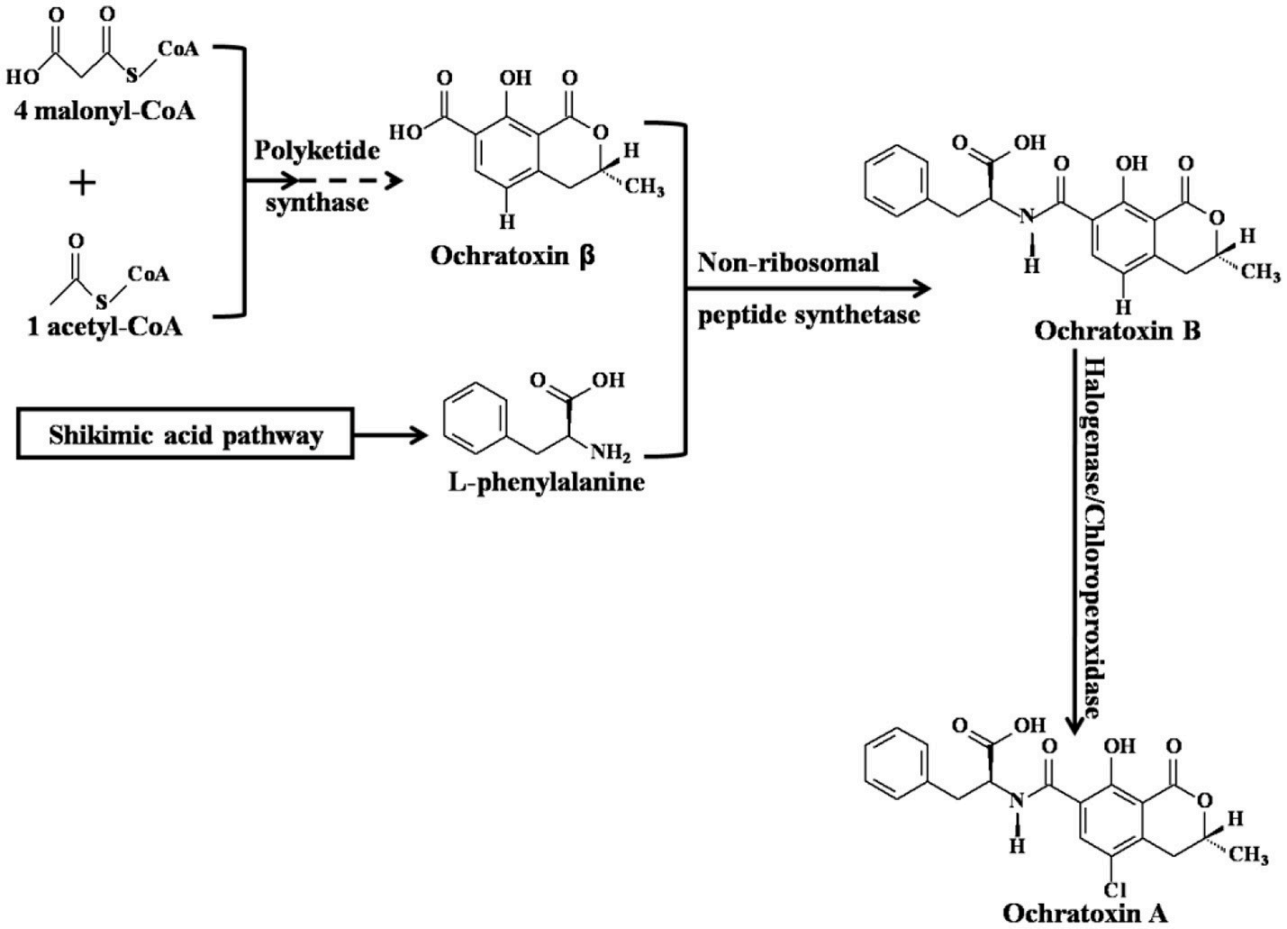
Critical steps of Ochratoxin A biosynthesis [adapted from (Gallo et al., 2017)].

Ochratoxin A (OTA), 7-carboxyl-5-chloro-8-hydroxyl-3,4-dihydro-3R-methyl-isocoumarin-7-L-β-phenylalanine (Figure 1; Wu et al., 2011), is a secondary fungal metabolite with low molecular weight that is mainly produced by various species of *Aspergillus* and *Penicillium* (Shen et al., 2013; Susca et al., 2016; Freire et al., 2017). Critical steps of OTA biosynthesis are shown in Figure 1 (Huff and Hamilton, 1979; Harris and Mantle, 2001; El Khoury and Atoui, 2010; Gallo et al., 2013, 2017; Wang et al., 2016). Common OTA-contaminated feeds (Streit et al., 2013; Li et al., 2014; Sherazi et al., 2015; Pinotti et al., 2016) and food commodities include cereals (maize, wheat, rice, sorghum, barley, oats, and rye) (Duarte et al., 2010; Liang et al., 2015; Lim et al., 2015; Sun et al., 2017), cereal products (bread, flour, and pasta) (Shen et al., 2014), wine (Quintela et al., 2013; Freire et al., 2017), dairy products (milk and cheese), meat (pork, fish, and poultry), eggs (Dai et al., 2014), fruit (grapes, apples, peaches, pears, strawberries, oranges, watermelons, mangoes, and figs) (Marin and Taranu, 2015; Freire et al., 2017), vegetables (yam, garlic, onions, potatoes, and tomatoes) (Pfohl-Leszkowicz and Manderville, 2007; Zhao et al., 2017), beans (coffee beans, peanuts, chickpeas, and soybeans) (Pfohl-Leszkowicz and Manderville, 2012; Wang et al., 2017), dried products (dried beans, smoked or salted dried fish, raisins, and jerky), nuts, sesame seeds, rapeseed, spice (Abrunhosa et al., 2010; Bui-Klimke and Wu, 2015), infant cereals (Cappozzo et al., 2017), and even milk-based baby formulae (Raiola et al., 2015; Zhao et al., 2017). According to the latest statistics from 2006 to 2016, the maximum concentration and incidence of OTA in raw cereal grains was 1,164 μg/kg and 29%, respectively (Lee and Ryu, 2017). Therefore, it is difficult to completely avoid the risk of OTA exposure. In 1993, OTA was classified as a group 2B carcinogen (possible human carcinogen) by the International Agency for Research on Cancer (IARC) (IARC, 1993). Although OTA has not been classified as a group 1 carcinogen (human carcinogen), such as aflatoxins, this does not mean that OTA is significantly less toxic than aflatoxins. In fact, OTA is a powerful carcinogen for rodents and poultry (Bondy et al., 2015), but the epidemiological evidence in human beings is very scarce (Bui-Klimke and Wu, 2015). That is to say, the carcinogenic evidence of OTA is not as sufficient as that of aflatoxins in human beings (Bui-Klimke and Wu, 2015). A great deal of animal or cell experiments have reported that the exposure of OTA can result in various toxicological effects, including the disruption of the gut microbiota homeostasis (Liew and Mohd-Redzwan, 2018), teratogenicity, carcinogenicity (Pfohl-Leszkowicz and Manderville, 2012), mutagenicity, hepatotoxicity (Zheng et al., 2013; Qi et al., 2014), genotoxicity (Pfohl-Leszkowicz and Manderville, 2007), immunotoxicity (Marin and Taranu, 2015), embryotoxicity (Hong et al., 2000), developmental toxicity, neurotoxicity (Bhat et al., 2016), testicular toxicity (Schwartz, 2002), blood-brain barrier damage (Jackson and Ryu, 2017), and especially nephrotoxicity (Zhao et al., 2017). The primary target organ of OTA is the kidney (Zhang et al., 2014). Although the human epidemiological evidence is inadequate, the association between OTA exposure and Balkan Endemic Nephropathy (BEN), Chronic Interstitial Nephropathy (CIN), and other kidney diseases is more or less existed (Bui-Klimke and Wu, 2015; Zhao et al., 2017). Thus, OTA attracts global concern based on its ubiquitous nature in feeds and foods and the adverse health effects in humans and animals (Duarte et al., 2010).

Apart from causing health issues, OTA exposure results in enormous economic losses via decreasing the productivity of livestock and farm crops and increasing the medical fees of humans and animals, the mortality rate of animals, and the costs related to OTA precaution, control, and detoxication (Duarte et al., 2010; Pfliegler et al., 2015). The best way to avoid these negative health and economic effects would be preventing the contamination of OTA. But in fact, it is nearly impossible to completely avoid its contamination in feeds and foodstuffs. Instead, to adopt various measures to detoxify OTA-contaminated foods and feeds is more feasible and necessary (Russo et al., 2016). According to present reports, physical, chemical, and biological detoxification methods were mainly applied to food and feed industries (Kabak and Dobson, 2009; Quintela et al., 2013). Among these three classes of methods, biological methods, with better safety, flavor, nutritional quality, organoleptic properties, availability, and cost-effectiveness, were more promising than the other two classes (Kabak and Dobson, 2009; Rodriguez et al., 2011; Hathout and Aly, 2014; De Bellis et al., 2015; Cho et al., 2016; Farbo et al., 2016). Thus, this review principally focuses on the advances in biodetoxification of OTA.

## Biodetoxification of OTA by degradation

Microorganisms, including actinobacteria, bacteria, filamentous fungi, and yeast, that are able to degrade OTA are summarized in Table S1. Note that the direct *in vivo* experiments with animals and the *in vitro* experiments with animal tissues or body fluids that do not have specific microorganisms separated are not summarized in Tables S1, S2, such as the research of Xiao et al. (1991); Madhyastha et al. (1992); Özpinar et al. (1999); Müller et al. (1998), and Müller et al. (2001). In addition, microorganisms that could detoxify OTA but without explicit adsorption and/or biodegradation mechanisms of OTA elimination were neither shown in Tables S1, S2, such as the research of Böhm et al. (2000); Štyriak et al. (2001), and Caridi et al. (2006).

### OTA biodegradation products and their properties

**Figure 2:**
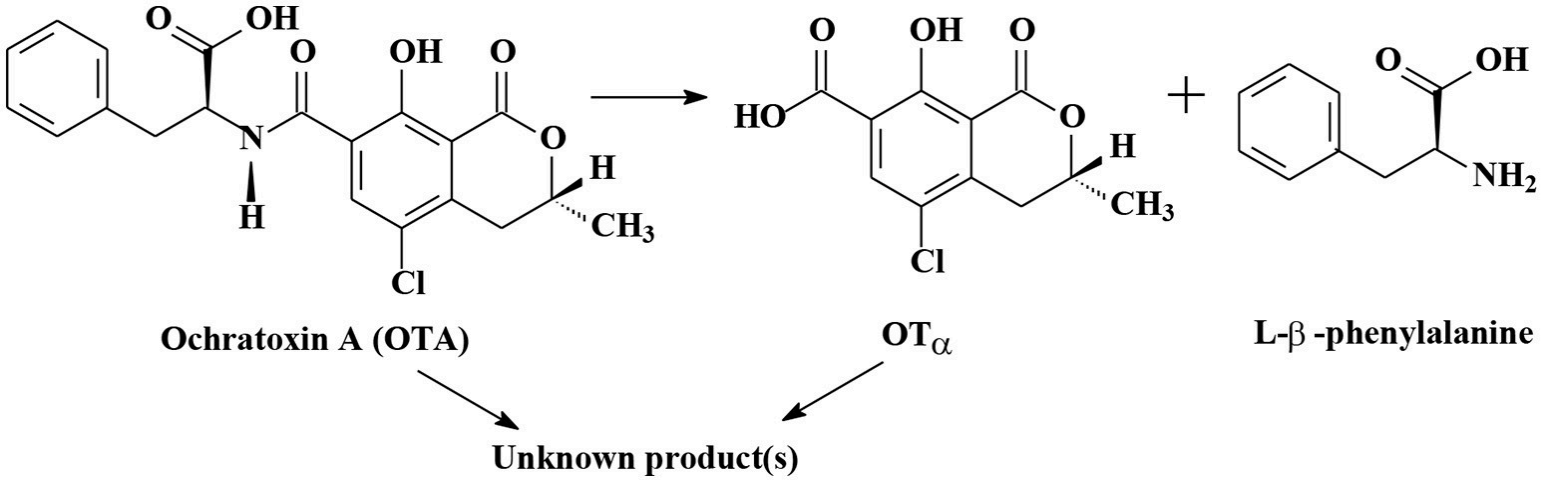
The biodegradation mechanisms of Ochratoxin A.

As shown in Table S1, the degradation of OTA to OTα (7-carboxy-5-chloro-8-hydroxy-3,4-dihydro-3-R-methylisocoumarin) is the most important mechanism of OTA biodegradation (Figure 2; Loi et al., 2017). Degradation products OTα and L-β-phenylalanine are formed by the hydrolysis of the amide bond via hydrolytic enzymes, such as carboxypeptidase A, carboxypeptidase PJ_1540, protease A, lipase A, ochratoxinase, etc. (Stander et al., 2000; Abrunhosa et al., 2006; Dobritzsch et al., 2014; Liuzzi et al., 2016).

Rodrigues et al. (2009) reported that OTα was non-toxic, or at least 500 times less toxic than OTA. The research of Ferenczi et al. (2014) revealed that the administration of OTA (1 or 10 mg/kg BW for 72 h or 0.5 mg/kg BW for 21 d) to male CD1 mice via oral gavage led to a significant increase of OTA concentration in the blood, transcriptional alterations in OTA-dependent genes (*gadd45, gadd153, sulphotransferase, annexinA2*, and *clusterin*) and histopathological changes in the renal cortex. These OTA-induced alterations in male CD1 mice were not observed in the group which OTA was degraded into OTα by treating OTA with *Cupriavidus basilensis* OR16 for 5 days (Ferenczi et al., 2014). Rodriguez et al. (2011) verified that 0.04 μg/mL of OTA was converted completely to OTα by food-borne *Brevibacterium* spp. strains (*B. casei* DSM 20657, DSM 9657, DSM 20658, RM101; *B. linens* DSM 20425; *B. iodinum* DSM20626; *B. epidermidis* DSM 20660) within 10 d. The tested strain, *B. casei* RM101, could completely cleave OTA even at 40 μg/mL, which is 1,000 times greater than the OTA concentration commonly found in foodstuffs (Rodriguez et al., 2011). Bejaoui et al. (2006) reported that OTα was further degraded into unknown products. *Aspergillus niger* GX312, *A. japonicus* AX35, and *A. carbonarius* SA332 (a weak OTA producer) could convert OTA to OTα by 99, 89, and 83% within 5 days, respectively. Then OTα was converted to an unknown compound after 9 days incubation (Bejaoui et al., 2006). A small amount of authors reported that OTA could be directly degraded into unknown products (Patharajan et al., 2011; Shi et al., 2013, 2014).

### Effects of different culture conditions on OTA biodegradation

The degradation of OTA by the same microorganism in different culture media has different manifestations (Varga et al., 2000, 2005; Abrunhosa et al., 2014). It is worth mentioning that an atoxigenic *A. niger* CBS 120.49 could completely degrade OTA (2.5 μg/mL) to OTα in solid medium within 5 d, faster than in liquid medium within 7 days (Varga et al., 2000). The degradation product, OTα, was further decomposed to an unknown compound within 7 days in solid media (Varga et al., 2000). Among 55 isolated *Rhizopus* and *Mucor* strains, many *Rhizopus* strains were able to degrade OTA (7.5 μg/mL) to a detection limit below concentration in liquid medium within 10 days (Varga et al., 2005). *A. niger* CBS 120.49 was able to degrade more than 90% of OTA within 6 d, while *R. stolonifer* var. *stolonifer* TJM 8A8 needed 12 days to degrade about 90% of OTA in liquid medium. Only *R. stolonifer* var. *stolonifer* TJM 8A8 was able to degrade 96.5% of OTA (7.5 μg/g) in moistened wheat during 10 days incubation (Varga et al., 2005). *Pediococcus parvulus* UTAD 473 was able to degrade 90% of OTA (1 μg/mL) within 25 h in MRS broth medium, while only degrading 80% of OTA (7 μg/L) after 6 days incubation in grape must. Furthermore, no obvious degradation of OTA (7 μg/L) was observed in synthetic wine (Abrunhosa et al., 2014).

Oxygen is also one of the important factors that affect the growth and reproduction of microorganisms. In addition to certain aerobic microorganisms, some anaerobic microorganisms were also able to degrade OTA (Schatzmayr et al., 2006; Upadhaya et al., 2012). Anaerobic *Eubacterium biforme* MM11, isolated from swine gut, was able to degrade 77.1% of OTA (0.1 μg/mL) in modified M 98-5 liquid medium within 12 h at 39°C. This strain could completely degrade 1 μg/mL of OTA in solid corn substrate within 24 h at 39°C, which suggests that anaerobic microorganisms might be suitable for the development of feed additives that will function in the targeted animal intestines. It is also worth noting that 26% of the OTA (1 μg/mL) was removed in the negative control (corn) within 24 h at 39°C (Upadhaya et al., 2012). Moreover, anaerobic *E. callanderi* Due4_11 was able to degrade 95% of OTA (0.2 μg/mL) to OTα within 6 h (Schatzmayr et al., 2006).

### Dually functional strains of OTA biodegradation

Some microorganisms were not only able to degrade OTA, but also able to inhibit the biosynthesis of OTA. El Khoury et al. (2017) reported that several actinobacterial strains (*Streptomyces* AT10, AT8, SN7, MS1, ML5, G10, PT1) were able to degrade 22.83–52.68% of OTA (0.095 μg/mL) within 5 d. At the same time, *Streptomyces* AT10, AT8, SN7, G10, and PT1 could adsorb 16.07%-33.93% of OTA (0.045 μg/mL) within 1 h. Furthermore, the expression of biosynthesis genes (*acOTAnrps, acpks*, and *acOTApks*) and regulatory gene (*veA*) of OTA in *A. carbonarius* S402 was inhibited by *Streptomyces* MS1, ML5, and G10, with the down regulation of 21.0, 11.9, and 11.9% for *acOTAnrps*, 37.1, 39.0, and 9.0% for *acpks*, 23.9, 23.0, and 18.3% for *acOTApks*, and 11.4, 0.0, and 7.0% for *veA*, respectively (El Khoury et al., 2017).

Some microorganisms have dual functions of OTA degradation and adsorption (Péteri et al., 2007; Shi et al., 2014). Shi et al. (2014) reported that the cell-free supernatant of *Bacillus subtilis* CW 14 could degrade 97.6% of OTA (6 μg/mL) within 24 h, but no degradation product was detected. Furthermore, 66.6 and 87.9% of OTA (6 μg/mL) was adsorbed by viable and dead *B. subtilis* CW 14 cells, respectively (Shi et al., 2014). Péteri et al. (2007) verified that *Phaffia rhodozyma* CBS 5905 was able to degrade 90% of OTA (7.5 μg/mL) within 15 d and adsorb 23% of OTA (3 μg/mL) within 2 h, respectively.

## Biodetoxification of OTA by adsorption

Microorganisms, including actinobacteria, bacteria, filamentous fungi, and yeast, that were able to adsorb OTA are summarized in Table S2. The most important influence factor of OTA adsorption capacity of microorganisms is cell wall components. However, there are controversies among different scholars on specific cell wall components, such as glucogalactans and β-glucans (Ben Taheur et al., 2017), mannoproteins (Caridi et al., 2012), β-glucans and mannans (Pereyra et al., 2015).

### Effects of different culture conditions on OTA adsorption

The adsorption of OTA by the same microorganism in different culture conditions has different manifestations (Armando et al., 2012; Piotrowska et al., 2013; Ben Taheur et al., 2017). Piotrowska et al. (2013) certified that *Saccharomyces cerevisiae* Syrena LOCK 0201 and *S. cerevisiae* Malaga LOCK 0173 removed 85.1 and 82.8% of OTA (1 μg/mL) in white grape juice as well as 65.2 and 10.7% of OTA (1 μg/mL) in blackcurrant juice after 10 days incubation, respectively. Armando et al. (2012) simulated mammalian gastrointestinal conditions, Yeast Peptone Dextrose (YPD) broth (pH 2 and pH 7) and YPD with 0.5% bile salts (pH 7), to study the effect of *S. cerevisiae* RC008, RC009, RC012, and RC016 on OTA (100 μg/mL) adsorption. Results proved that the OTA binding level of *S. cerevisiae* RC008 and RC009 was 82.3 and 80.2% in YPD broth (pH 2), 74.4 and 78.7% in YPD with 0.5% bile salts (pH 7), 56.7 and 67.1% in YPD broth (pH 7), respectively. The OTA binding ability of *S. cerevisiae* RC008 and RC009 was significantly stronger in simulated mammalian gastrointestinal conditions than that in YPD broth (pH 7), while the OTA binding ability of *S. cerevisiae* RC012 and RC016 was not significantly different between simulated mammalian gastrointestinal conditions and YPD broth (pH 7). Mammalian gastrointestinal conditions enhanced the adsorption of *S. cerevisiae* to OTA, or at least did not reduce OTA-*S. cerevisiae* interactions (Armando et al., 2012). Ben Taheur et al. (2017) reported that *Acetobacter syzygii* KFGM1 and *Lactobacillus kefiri* KFLM3 were able to bind 15 and 15% of OTA in Man Rogosa Sharpe (MRS) medium while 50 and 81% of OTA in milk during 1 d incubation, respectively. *Kazachstania servazzii* KFGY7 could adsorb 6 and 62% of OTA in yeast extract peptone dextrose (YPD) and in milk during 1 d incubation, respectively. The reason might be that the nutrient components of milk were better than those of MRS or YPD, which facilitated the growth of microorganisms and the biosynthesis of cell wall ingredients concerning OTA adsorption. The OTA adsorption mechanism of *L. kefiri* KFLM3 and *K. servazzii* KFGY7 was possibly involved in the function of hydrosoluble glucogalactan exopolysaccharides and β-(1,3 and 1,6)-D-glucans of the cell wall, respectively (Ben Taheur et al., 2017).

### Effects of different statuses on OTA adsorption

The adsorption of OTA by the same microorganism but in different statuses (viable/dead) has similar or different manifestations (Bejaoui et al., 2004, 2005; Péteri et al., 2007; Mateo et al., 2010; Fiori et al., 2014; Piotrowska, 2014). Péteri et al. (2007) reported that viable and dead *Phaffia rhodozyma* CBS 5905 were able to remove 23 and 45% of OTA (3 μg/mL) within 2 h, respectively. Mateo et al. (2010) demonstrated that the OTA adsorption capacity of *Oenococcus oeni* 6G and *O. oeni* 124 M was not significantly different between viable and dead cells after 30 min incubation with 2 μg/L of OTA. Fiori et al. (2014) tested the OTA adsorption capacity of viable/dead yeast strains. Results demonstrated that viable and dead *Candida friedrichii* 778, *Candida intermedia* 235, *Lachancea thermotolerans* 751, *Cyberlindnera jadinii* 273 could adsorb 70, 73, 75, and 0% of OTA (0.02 μg/mL) and 72, 74, 84, and 82% of OTA within 8 days, respectively. The OTA adsorption capacity varied greatly between viable and dead yeast strain *C. jadinii* 273 (Fiori et al., 2014). Bejaoui et al. (2005) reported that the OTA adsorption ability of dead *A. niger* GX312, *A. carbonarius* SA332 (a weak OTA producer), and *A. Japonicus* AX35, with the OTA (2 μg/mL) reduction of 47.5, 66.5, and 41.5% within 2 h, was at least 10% higher than that of viable *A. niger* GX312, *A. carbonarius* SA332 (a weak OTA producer), and *A. Japonicus* AX35, with the OTA (2 μg/mL) reduction of 30, 55, and 30% within 2 h. While viable or dead *A. niger* GX312, *A. carbonarius* SA332 (a weak OTA producer), and *A. Japonicus* AX35 displayed the same adsorption ability to low concentration of OTA that 80% of OTA (0.01 μg/mL) was removed within 2 h (Bejaoui et al., 2005). Bejaoui et al. (2004) demonstrated that viable and dead *S. cerevisiae* LALVIN Rhône 2056 was able to remove 17 and 75% of OTA (2 μg/mL) within 2 h in liquid yeast peptone glucose (YPG) medium, respectively. Furthermore, the OTA adsorption ability was not significantly influenced by different treatment methods that lead to microbial cell death, since no significant difference was observed between heat and acid treatments of *S. cerevisiae* LALVIN Rhône 2056 cells. Heat treatment resulted in the formation of Maillard reaction products, the denaturation of proteins, the reduction of cell wall thickness of peptidoglycan, or the enlargement of pore size, which may be responsible for more exposure of OTA adsorption sites than viable cells. Acid treatment led to the formation of monomers and aldehydes products via breaking down polysaccharides, the reduction of cell wall thickness of peptidoglycan, or the enlargement of pore size, which may be responsible for more exposure of OTA adsorption sites as well (Bejaoui et al., 2004). Piotrowska (2014) verified that the dead *Lactobacillus plantarum* LOCK 0862, *L. brevis* LOCK 0845, and *L. sanfranciscensis* LOCK 0866 could reduce 46.29–59.82% of OTA (1 μg/mL) within 30 min in PBS buffer, but the alive *L. plantarum* LOCK 0862, *L. brevis* LOCK 0845, and *L. sanfranciscensis* LOCK 0866 needed 24 h to remove 14.80–26.42% of OTA (1 μg/mL) in PBS buffer. The adsorption of OTA to the surface structures of the lactic acid bacteria cell wall is influenced by hydrophobic, acceptor/acidic, and donor/basic properties of the cell surface (Piotrowska, 2014).

### Effects of microbial cell wall components on OTA adsorption

In addition, some researchers have specifically studied the OTA adsorption ability of microbial cell walls (Pereyra et al., 2015). Two commercial yeast cell walls (YCW) with different β-glucans/mannans percentages, YCW1 (23.3% of polysaccharides containing 17.4% of β-glucans and 5.9% of mannans) and YCW2 (44.0% of polysaccharides containing 23.0% of β-glucans and 21.0% of mannans), were used to evaluate their OTA adsorption ability in simulated stomachal conditions of monogastric animals by Pereyra et al. (2015). Results proved that the OTA adsorption ability was not significantly influenced by different percentages of polysaccharides or β-glucans/mannans (Pereyra et al., 2015).

### Effects of strains and their statuses on the firmness of OTA adsorption

The firmness of OTA adsorption was strain-specific (Caridi et al., 2012; Petruzzi et al., 2015). Petruzzi et al. (2015) reported that *S. cerevisiae* W28 and W46 was able to bind 34.51–70.17% and 42.79–76.44% of OTA (2 μg/L) in grape must, respectively. But, the binding of *S. cerevisiae*-OTA was reversible with 80%-85% of the initially binding OTA releasing back into the washing solution. The binding ability of *S. cerevisiae*-OTA complex varied among different kinds of strains. The *S. cerevisiae* W13 could remove 30.69–53.79% of OTA (2 μg/L) in grape must, but its releasing-back percentage was 55%, which was significantly lower than that of *S. cerevisiae* W28 and W46 (Petruzzi et al., 2015). Caridi et al. (2012) verified that there is a great difference between descendants and their parents (*S. cerevisiae* TP5 and TT173) in the homogeneity of OTA adsorption capacity. The different content of mannoproteins might contribute to the difference of OTA adsorption capacity of yeast cells, because OTA and mannoproteins were linked through ionic and electrostatic interactions (Caridi et al., 2012).

The firmness of OTA adsorption by the same microorganism in different statuses (viable/dead) was also different (Piotrowska, 2012). The bond between microbial cells and OTA was partially reversible. Piotrowska (2012) reported that the complex of viable cells-OTA was firmer than the complex of dead cells-OTA, with 11 and 22% of initially binding OTA releasing back into PBS buffer, respectively. Protoplasts of *S. cerevisiae* BS lost the capacity to bind OTA, indicating that the components of the cell wall were strongly associated with the adsorption of OTA (Piotrowska, 2012).

## Biodetoxification of OTA by enzymes

Enzymes, including crude and purified, that were able to cleave OTA, are summarized in Table S3.

The first report about the biodetoxification of OTA took place in 1969 (Pitout, 1969), which was only four years after the discovery of OTA (Van Der Merwe et al., 1965). It used bovine pancreatic carboxypetidase A (CPA) to cleave OTA to OTα (Pitout, 1969).

Abrunhosa et al. (2006) reported that Ancex, a crude enzyme isolated from *A. niger* MUM 03.58, was able to degrade 99.8% of OTA (1 μg/mL) to OTα after 25 h incubation at pH 7.5 and 37°C. Commercially purified enzymes did not degrade nearly as much. For example, 87.3 and 43.4% of OTA was degraded by Protease A and Pancreatin after 25 h incubation at pH 7.5 and 37°C, respectively (Abrunhosa et al., 2006). Furthermore, another commercially purified enzyme, Prolyve PAC, was able to degrade only 3% of OTA (1 μg/mL) to OTα after 25 h incubation at pH 3 (optimal pH) and 37°C (Abrunhosa et al., 2006). A crude metalloenzyme, with 12.8 times higher OTA hydrolytic activity than that of CPA at pH 7.5 and 37°C, was isolated from *A. niger* by Abrunhosa and Venâncio (2007). Finally, an OTA hydrolytic activity of 36 U/mg was obtained from the purified enzyme (Abrunhosa and Venâncio, 2007). Cho et al. (2016) reported that 97.5 and 91.3% of OTA (0.04 μg/mL) was removed by crude enzymes of *A. tubingensis* M036 and M074 at pH 5 and 25°C within 24 h, respectively.

Dobritzsch et al. (2014) reported that the purified recombinant ochratoxinase was about 600 times more efficiently hydrolyzed OTA than CPA at an optimal pH of CPA (pH 7.5). The optimal OTA-degrading activity of this novel ochratoxinase was obtained at 66°C and about pH 6 (Dobritzsch et al., 2014).

In general, although researchers have isolated many OTA biodegradable microorganisms, there is still a lack of isolation and purification of enzymes and performance studies among them. This may be mainly due to the relatively small number of interdisciplinary studies, and relatively few experts familiar with the relevant studies of both microorganisms and enzymes, thus limiting the subsequent in-depth study of abundant microorganisms with excellent biodegradability.

## Industrial application prospects of OTA biodetoxification

It has been reported that a large number of patented microorganisms have great application prospects in food and feed industries (Guan et al., 2009; Jiang et al., 2016a,b,c,d). After 72 h incubation with *Bacillus licheniformis* MZH-11, 0.1, 0.5, and 5 μg/g of OTA in corn flour was degraded by 84.4,78.3, and 73.5%, respectively (Guan et al., 2009). Although the concentration of OTA in corn flour increased by 10-fold from 0.5 to 5 μg/g, the decreased degradation rate of OTA from 78.3 to 73.5% was very little, which indicated the great application prospects of *B. licheniformis* MZH-11 in the feed industry (Guan et al., 2009). Jiang et al. (2016b) reported that 0.02 μg/g of OTA in corn-soybean feed was degraded by 71% after 72 h incubation with *Stenotrophomonas* sp. CW117. Moreover, 0.02 μg/g of OTA in corn-soybean feed was degraded by 48.3, 53.2, and 68.7% following 48 h incubation with *Luteimonas* sp. CW574 (Jiang et al., 2016d), *Silanimonas* sp. CW282 (Jiang et al., 2016c), and *Lysobacter* sp. CW239 (Jiang et al., 2016a), respectively.

It is essential for industrial application of strains to maintain good performance during processing, such as lyophilization. Schatzmayr et al. (2006) used an *in vitro* model with whole pieces of pig gut to evaluate the OTA-degradation activity of lyophilized powders of *Trichosporon mycotoxinivorans* (MTV, 115) and *Stenotrophomonas nitritreducens* 041-9 strains. Results revealed that the lyophilized powders of *T. mycotoxinivorans* (MTV, 115) or *S. nitritreducens* 041-9 could degrade about 90% of OTA (0.4 μg/mL) within 6 h, which displayed great potential to be applied as OTA-detoxifying feed additive (Schatzmayr et al., 2006). Due to the excellent OTA-detoxification performance of *T. mycotoxinivorans* (MTV, 115), it was made into a product named Mycofix® Plus^MTVINSIDE^ by Biomin GmbH (Austria) (Hanif et al., 2008). Hanif et al. (2008) added 1‰ and 2‰ Mycofix® Plus^MTVINSIDE^ (containing 6. 0 × 10^8^ count *T. mycotoxinivorans* MTV cells/g) to the feed containing OTA (0.5 and 1 μg/g) for studying the attenuating effects of Mycofix® Plus^MTVINSIDE^ on the undesirable effects of OTA in 1-day-old broiler chicks over a 42-days period. Results confirmed that OTA induced adverse effects, such as poor feed conversion ratio, depressed body weight gain, increased levels of serum enzymes (lactate dehydrogenase, aspartate aminotransferase, and γ-glutamyltranspeptidase) and significant histopathological changes in bursa of fabricius, spleen, liver, and kidney, were appreciably attenuated by Mycofix® Plus^MTVINSIDE^ (Hanif et al., 2008). Although Mycofix® Plus^MTVINSIDE^ has many advantages in OTA detoxification, *T. mycotoxinivorans* was recognized in 2009 as a possible novel human pathogen that is related to cystic fibrosis (Hickey et al., 2009), suggesting that the secondary metabolites of *T. mycotoxinivorans* are extremely complicated. Therefore, *T. mycotoxinivorans* deserves further investigations to reduce possible deleterious effects and should be used with caution in food or feed industries. It is noteworthy that the application of microorganisms to the food and feed industries must be cautious, with particular attention to risks for human health (Spano and Capozzi, 2011; Capozzi et al., 2017).

It has also been reported that many microorganisms with good OTA adsorption ability have great application prospects in food and feed industries (Moruno et al., 2005; Csutorás et al., 2013; Farbo et al., 2016). Csutorás et al. (2013) used macro-scale experiments (16 L glass balloons in wine cellar) to simulate a real wine industrial system. During a 90-day fermentation process, OTA (4 μg/mL) was adsorbed to *S. cerevisiae* by 73, 85, and 90% in white, rose, and red wine musts, respectively (Csutorás et al., 2013). Moruno et al. (2005) used *S. cerevisiae* 169-involving fermentation lees of red and white grape musts to treat OTA-containing red wine samples. Results certified that 4.12 and 7.09 μg/L of OTA was adsorbed by 58.7 and 71.4% in 80 d red wine fermentation with red lees and white lees, respectively (Moruno et al., 2005). Farbo et al. (2016) immobilized *Candida intermedia* 253 yeast cells into magnetic calcium alginate beads to adsorb OTA in commercial grape juice. Results showed that more than 80% of OTA (0.02 μg/g) was adsorbed within 48 h of incubation, while OTA was slowly released back into the commercial grape juice from calcium alginate beads in the following phases (72–120 h). Thus, the authors developed a glass chromatography column-containing prototype bioreactor encapsulating *C. intermedia* 253 yeast cells in calcium alginate beads to decontaminate OTA in liquid matrices. The concentration of OTA (0.02 μg/g) in commercial grape juice was reduced by 21 and 57% via the first column filtration step and the fourth column filtration step, respectively (Farbo et al., 2016). Furthermore, three industrial yeast by-products including EX16 (a vinasse containing 16% liquid yeast cell walls), LEC (a dry yeast cell wall fraction), and BETA (a dried purified β-glucans of yeast cell wall fraction) were used to adsorb OTA by Ringot et al. (2007). Both non-polar and polar non-covalent interactions were involved in the plentiful OTA adsorption onto LEC. The non-polar interactions concerned the interactions between hydrophobic amino acids of LEC and aromatic rings of OTA. The polar interactions could be interpreted by four different complementary patterns as follows: (a) electrostatic π-π interactions between aromatic amino acids of LEC and aromatic rings of OTA, (b) electrostatic ionic interactions between basic amino acids of LEC and carboxylic group of OTA, (c) hydrogen bond interactions between donor groups of LEC and receptor aromatic rings of OTA, (d) hydrogen bond interactions between donor groups (phenol and amide group of OTA) and receptor groups of LEC. The relative importance of these interactions between OTA and LEC still needs to be further explored (Ringot et al., 2005).

Furthermore, it has been reported that many microorganisms could detoxify OTA, but the adsorption and/or biodegradation mechanisms of OTA elimination were not reported (not shown in Tables S1–S3). Kapetanakou et al. (2012) applied strains mixtures (10^7^ cfu/mL) with 16 yeasts isolated from different batches of wine and 29 bacteria isolated from fermented flour, basil, sourdough, sausage, and aniseed to degrade OTA (0.1 μg/mL) in grape juice, red wine, and beer at 25°C for 5 days. Results shown OTA reduction was 32, 22, and 12% in grape juice, red wine, and beer, respectively (Kapetanakou et al., 2012). The licensed probiotic preparation, including *L. paracasei* LOCK 0920, *L. plantarum* LOCK 0945, *L. brevis* LOCK 0944, *S. cerevisiae* LOCK 0140, and *Yucca schidigera* extract, was applied to reduce OTA in broiler feed by Slizewska and Piotrowska (2014). The 1 and 5 mg/kg of OTA was reduced by 73 and 55% in feed after 6 h fermentation with the probiotic preparation, respectively. At the same time, aerobic spore-bearing bacteria were inhibited by the probiotic preparation (Slizewska and Piotrowska, 2014). Cai et al. (2014) patented a yeast strain isolated from pig feces and nearby soil, *Kluyveromyces marxianus* C2, which could reduce 82.3% of OTA (0.5 μg/mL) in YPD medium and 83.7% of OTA (0.082 μg/g) in moldy corn feed, respectively.

In addition, it has been reported that a number of enzymes originating from some microorganisms with good OTA degradation ability have great application prospects in food and feed industries (Abrunhosa et al., 2006; Yu et al., 2015). Amidase 2, a patented food or feed additive, was cloned from *A. niger* to degrade OTA to OTα by Yu et al. (2015). After 2.5 h incubation with amidase 2 at 40°C, the content of OTA was reduced from 47 ng/mL to less than 2 ng/mL in milk (Yu et al., 2015). After 20 h incubation with amidase 2, 38 μg/mL of OTA was reduced to less than 2 ng/mL in corn flour and to 6.6 μg/mL in corn soy based feed, respectively (Yu et al., 2015). Because of the low toxigenicity, *A. niger* is known as an GRAS (generally regarded as safe) strain, which, to a certain degree, indicates that *A. niger*-derivatived enzymes have great safety (Varga et al., 2000). Abrunhosa et al. (2006) reported that Ancex, a crude enzyme isolated from *A. niger* MUM 03.58, was able to degrade 87.9% of OTA (1 μg/mL) to OTα after 3 h incubation at pH 7.5 and 50°C.

## Future directions

Since the discovery of which carboxypeptidase A could degrade OTA in 1969, studies on OTA biodetoxification have been carried out for nearly 50 years. However, there are still many problems that remain to be solved. For example, compared to a single-OTA exposure environment, is the biodetoxification of strains equally effective in a multimycotoxins environment? Moreover, is the strain equally effective in a variety of food or feed complex systems? Furthermore, is it possible to screen a large number of strains in pre-harvest period to prevent OTA biosynthesis and in post-harvest period to biodetoxify OTA at the same time? In addition, at present, the vast majority of studies have been confined to the traditional isolation and screen of strains from different sources. It is necessary to make better use of transgenic technology in mutant strains to obtain better biodetoxification performance of OTA or OTA combined with several different mycotoxins. As the prevailing situation is that a myriad of mycotoxins coexist in the food or/and feed systems, obtaining high-performance strains that simultaneously biodegrade or/and adsorb several mycotoxins is bound to be a trend in the future. Of course, achieving these must be based on the clear studies concerning the biodetoxification mechanisms and genes of several different mycotoxins.

## Conclusion

On the basis of the development of about 50 years, researchers have identified a good deal of microorganisms, including actinobacteria, bacteria, filamentous fungi, and yeast, that could degrade and/or adsorb OTA. The degradation of OTA to non-toxic or less toxic OTα is the most important OTA biodegradation mechanism. The OTA adsorption capacity of microorganisms is possibly influenced by the glucogalactan exopolysaccharide, β-(1,3 and 1,6)-D-glucans, mannoproteins, and mannans of the cell wall and the hydrophobic, acceptor/acidic, and donor/basic properties of the cell surface via ionic and electrostatic interactions. The formation of Maillard reaction products, monomers and aldehydes products, the denaturation of proteins, the reduction of cell wall thickness of peptidoglycan, or the enlargement of pore size of dead cells (heat or acid treatment) resulted in more exposure of OTA adsorption sites than that of viable cells, which might be responsible for higher OTA adsorption. Numerous OTA degradation enzymes were isolated or cloned from bacteria, filamentous fungi, yeast, and animal pancreas. A large number of microorganisms with good OTA degradation and/or adsorption ability, as well as some OTA degradation enzymes, have great application prospects in food and feed industries. It is noteworthy that the application of microorganisms to the food and feed industries must be cautious, with particular attention to their safety.

## Author contributions

WC and CL wrote the manuscript. JY, ZZ, and XL searched the databases. BZ offered suggestions and polished language. YS, XhL, YW, and YL offered suggestions. XS modified the article.

### Conflict of interest statement

The authors declare that the research was conducted in the absence of any commercial or financial relationships that could be construed as a potential conflict of interest.
