# Supplementary material for: Advances in Biodetoxification of Ochratoxin A-A Review of the Past Five Decades
Source: Front Microbiol. 2018 Jun 26;9:1386. doi: 10.3389/fmicb.2018.01386 (PMC6028724; doi:10.3389/fmicb.2018.01386)
Supplement: Supplementary file 1 [file Table_1.pdf]

Table S1 Summary of OTA degrading microorganisms

Microorganism type	Species/strain	Source	OTA concentration(μg/mL)	Incubation time	Degradation rate (%)	Degradation products	Degradation enzyme	Reaction conditions	Reference
Actinobacteria	*Streptomyces* AT10, AT8, SN7, MS1, ML5, G10, PT1	soil	0.095	5d	22.83-52.68	NR	NR	liquid laboratory medium	El Khoury et al. (2017)
Bacteria	*Bacillus licheniformis* Sl-1[a]	animal faeces	6.2	24h	98	unknown	NR	liquid laboratory medium	Shi et al. (2013)
Bacteria	*Bacillus licheniformis* Sl-1	animal faeces	NR	72h	35	unknown	NR	moldy corn	Shi et al. (2013)
Bacteria	*Phenylobacterium immobile*	soil	100	3-5h	NR	OTα	dihydrodioldehydrogenase	liquid laboratory medium	Wegst and Lingens (1983)
Bacteria	*Acinetobacter calcoaceticus* strain 396.1	Vineyard soils	1	6d	82	OTα	NR	liquid laboratory medium	De Bellis et al. (2015)
Bacteria	*Acinetobacter* sp. neg1	Vineyard soils	1	6d	91	OTα	NR	liquid laboratory medium	De Bellis et al. (2015)
Bacteria	*Acinetobacter* sp. neg1 [a]	vineyard soil	1	6d	>70	OTα	β-metallo-lactamase (possible)	liquid laboratory medium	Fanelli et al. (2015)
Bacteria	*Pediococcus parvulus* UTAD 473	Douro wines	1	25h	90	OTα	NR	liquid laboratory medium	Abrunhosa et al. (2014)
Bacteria	*Pediococcus parvulus* UTAD 473	Douro wines	0.007	6d	80	OTα	NR	grape must	Abrunhosa et al. (2014)
Bacteria	*Lactobacillus plantarum*, *L. sanfrancisco*, *L. brevis*	BioStarPlus Company (Poland)	0.3 [c]	24h	54, 50, 37	NR	NR	liquid laboratory medium	Piotrowska and Zakowska (2000)

Microorganism type	Species/strain	Source	OTA concentration(μg/mL)	Incubation time	Degradation rate (%)	Degradation products	Degradation enzyme	Reaction conditions	Reference
Bacteria	*Acinetobacter calcoaceticus* NRRL B-551	Northern Regional Agricultural Research Service of United States Drug Administration	10	5d	100	OTα	NR	liquid laboratory medium	Hwang and Draughon (1994)
Bacteria	*Bacillus amyloliquefaciens* ASAG1 [a]	depot-stored maize	1	24h	98.5	OTα	carboxypeptidase	liquid laboratory medium	Chang et al. (2015)
Bacteria	*Brevibacterium casei* DSM 20657, DSM 9657, DSM 20658, RM101; *B. linens* DSM 20425; *B. iodinum* DSM20626; *B. epidermidis* DSM 20660	German Collection of Microorganisms and Cell Cultures	0.04	10d	100	OTα	carboxypeptidase A (possible)	liquid laboratory medium	Rodriguez et al. (2011)
Bacteria	*Brevibacterium casei* RM101	German Collection of Microorganisms and Cell Cultures	40	10d	100	OTα	carboxypeptidase A (possible)	liquid laboratory medium	Rodriguez et al. (2011)
Bacteria	*Lactobacillus acidophilus* VM 20	University of Veterinary Medicine (Vienna, Austria)	1	4h	96	NR	NR	liquid laboratory medium	Fuchs et al. (2008)
Bacteria	*Bacillus licheniformis*	rumen fluid of goat	0.1	6h	68	NR	carboxypeptidase A	roughage	Upadhaya et al. (2011)
Bacteria	*Alcaligenes faecalis* ASAGF 0D-1	soil	1	48h	92	OTα	carboxypeptidase A (possible)	liquid laboratory medium	Zhang et al. (2017)
Bacteria	*Bacillus licheniformis* CM 21	Thai fermented soybean product (Thua-nao)	5	2d	92.5	OTα	NR	liquid laboratory medium	Petchkongkaew et al. (2008)
Bacteria	*Eubacterium biforme* MM11 [b]	swine intestinal microbiota	0.1	12h	77.1	NR	NR	liquid laboratory medium	Upadhaya et al. (2012)

Microorganism type	Species/strain	Source	OTA concentration(μg/mL)	Incubation time	Degradation rate (%)	Degradation products	Degradation enzyme	Reaction conditions	Reference
Bacteria	*Eubacterium biforme* MM11 [b]	swine intestinal microbiota	1	24h	100[d]	NR	NR	solid corn substrate	Upadhaya et al. (2012)
Bacteria	*Eubacterium callanderi* Due4_11 [b]	pig intestine	0.2	6h	95	OTα	NR	liquid laboratory medium	Schatzmayr et al. (2006)
Bacteria	*Sphingomonas paucimobilis* 033-1, *S. asaccharolytica* 034-1	soil	0.2	5h	>95	OTα	NR	liquid laboratory medium	Schatzmayr et al. (2006)
Bacteria	*Stenotrophomonas nitritreducens* 041-9	soil	0.2	10h	100	OTα	NR	liquid laboratory medium	Schatzmayr et al. (2006)
Bacteria	*Rhodococcus erythropolis* GD2A, BRB 1AB	Natural soil	2	72h	27-34	NR	NR	liquid laboratory medium	Cserháti et al. (2013)
Bacteria	*Rhodococcus pyridinivorans* K402, K408	Oil contaminated soil	2	72h	15-21	NR	NR	liquid laboratory medium	Cserháti et al. (2013)
Bacteria	*Cupriavidus basilensis* ŐR16	soil	20	5d	100	OTα	NR	liquid laboratory medium	Ferenczi et al. (2014)
Bacteria	*Bacillus subtilis* CW 14 [a]	fresh elk droppings	6	24h	97.6	unknown	NR	PBS buffer	Shi et al. (2014)
Bacteria	*Bacillus subtilis* CW 14	fresh elk droppings	100 [c]	3d	47.1	unknown	NR	contaminated maize	Shi et al. (2014)
Bacteria	*Brevundimonas vermicularis* B-1	Vineyard soil	1	2d	87	NR	NR	liquid laboratory medium	Wang et al. (2014)
Bacteria	*Bacillus licheniformis* MZH-11	animal faeces et al.	5 [c]	36h	73.6	NR	NR	liquid laboratory medium	Guan et al. (2009) [e]
Bacteria	*Bacillus licheniformis* MZH-11	animal faeces et al.	0.1,0.5,5 [c]	72h	84.4,78.3, 73.5	NR	NR	corn flour	Guan et al. (2009) [e]

Microorganism type	Species/strain	Source	OTA concentration(μg/mL)	Incubation time	Degradation rate (%)	Degradation products	Degradation enzyme	Reaction conditions	Reference
Bacteria	*Stenotrophomonas* sp. CW117	soil and moldy food	0.02	72h	99.4	NR	NR	liquid laboratory medium	Jiang et al. (2016b) [e]
Bacteria	*Stenotrophomonas* sp. CW117	soil and moldy food	0.02 [c]	72h	71	NR	NR	feed	Jiang et al. (2016b) [e]
Bacteria	*Luteimonas* sp. CW574	soil and moldy food	0.02	48h	90.1	NR	NR	liquid laboratory medium	Jiang et al. (2016d) [e]
Bacteria	*Luteimonas* sp. CW574	soil and moldy food	0.02 [c]	48h	48.3	NR	NR	feed	Jiang et al. (2016d) [e]
Bacteria	*Silanimonas* sp. CW282	soil and moldy food	0.02	48h	95.6	NR	NR	liquid laboratory medium	Jiang et al. (2016c) [e]
Bacteria	*Silanimonas* sp. CW282	soil and moldy food	0.02 [c]	48h	53.2	NR	NR	feed	Jiang et al. (2016c) [e]
Bacteria	*Lysobacter* sp. CW239	soil and moldy food	0.02	48h	99.8	NR	NR	liquid laboratory medium	Jiang et al. (2016a) [e]
Bacteria	*Lysobacter* sp. CW239	soil and moldy food	0.02 [c]	48h	68.7	NR	NR	feed	Jiang et al. (2016a) [e]
Bacteria	*Pseudomonas aeruginosa* N17-1	soil	1	72h	85.4	NR	NR	liquid laboratory medium	Liu et al. (2016) [e]
Bacteria	*Bifidobacterium bifidum* CECT 870T, *B. breve* CECT 4839T; *Lactobacillus casei* CECT 475T, *Lactobacillus casei* CECT 4040, *L. casei* CECT 4045, *L. delbrueckii bulgaricus* CECT 4005, *L. johnsonii* CECT 289, *L. paracasei* CECT 4022, *L. plantarum* CECT 220, *L. plantarum* CECT 221, *L. plantarum* CECT 222, *L. plantarum* CECT 223, *L. plantarum* CECT 748, *L. plantarum* CECT 749, *L. rhamnosus* CECT 278T, *L. rhamnosus* CECT 288, *L. salivarius* CECT 4062[b]	Spanish Type Culture Collection	0.6	24h	29.6-97.1	OTα	NR	liquid laboratory medium	Luz et al. (2018)

Microorganism type	Species/strain	Source	OTA concentration(μg/mL)	Incubation time	Degradation rate (%)	Degradation products	Degradation enzyme	Reaction conditions	Reference
Filamentous fungi	*Aspergillus niger* GX312, *A. Japonicus* AX35	French grapes	2	5d	99, 89	OTα	NR	liquid laboratory medium	Bejaoui et al. (2006)
Filamentous fungi	*Aspergillus carbonarius* SA332 (a weak OTA producer)	French grapes	2	5d	83	OTα	NR	liquid laboratory medium	Bejaoui et al. (2006)
Filamentous fungi	Aspergillus tubingensis M036, M074	Korean meju (a starter of soybeans fermentation)	0.04	14d	>95	OTα	NR	liquid laboratory medium	Cho et al. (2016)
Filamentous fungi	*A. niger*, *A. carbonarius*, *A. fumigatus*, *A. clavatus*, *A. ochraceus*, *A. versicolor*,*A. wentii*, *Cladosporium* sp., *Penicillium aurantiogriseum*, *Penicillium spinulosum*	Portuguese grapes	1	6d	>80	OTα	carboxypeptidase A (possible)	liquid laboratory medium	Abrunhosa et al. (2002)
Filamentous fungi	*Botrytis cinerea* UdLTA 3·95, UdLTA 3·102, UdLTA 3·115	grapes	1	7d	24.2-26.7	NR	NR	solid grape synthetic medium	Valero et al. (2008)
Filamentous fungi	*Pleurotus ostreatus*	purchase	0.2 [c]	4w	77.3	OTα	NR	barley solid state fermentation medium	Engelhardt (2002)
Filamentous fungi	*Rhizopus stolonifer*, *R. microsporus*, *R. homothallicus*, *R. oryzae*, unidentified *Rhizopus sp.*	peach, nectarine, tomato, soil, rice, tempeh	7.5	16d	>95	OTα	carboxypeptidase A (possible)	liquid laboratory medium	Varga et al. (2005)
Filamentous fungi	*R. stolonifer* var. *stolonifer* TJM 8A8	peach	7.5 [c]	10d	96.5	OTα	carboxypeptidase A (possible)	moistened wheat	Varga et al. (2005)
Filamentous fungi	*Aspergillus niger* CBS 120.49	Central Bureau of Fungal Cultures (Netherlands)	2.5	7d,5d	100	OTα	NR	liquid and solid media	Varga et al. (2000)
Filamentous fungi	*Aspergillus niger* M00120	soil	0.25	2d	99	OTα	NR	liquid laboratory medium	Xiong et al. (2017)

Microorganism type	Species/strain	Source	OTA concentration(μg/mL)	Incubation time	Degradation rate (%)	Degradation products	Degradation enzyme	Reaction conditions	Reference
Filamentous fungi	*Aureobasidium pullulans* AU14-3-1, AU18-3B, AU34-2, LS30	Apple leaves, Plum fruits, Grapevine leaves, Apple fruits	0.8	6d	75-90.5	OTα	NR	liquid laboratory medium	de Felice et al. (2008)
Filamentous fungi	*Aureobasidium pullulans* AU14-3-1, AU18-3B	Apple leaves, Plum fruits	0.8	6d	25, 31	OTα	NR	grape must	de Felice et al. (2008)
Yeast	*Saccharomyces cerevisiae*	BioStarPlus Company (Poland)	0.3	24h	41	NR	NR	liquid laboratory medium	Piotrowska and Zakowska (2000)
Yeast	*Kazachstania servazzii* KFGY7	Kefir grains	1	24h	11	NR	NR	milk	Ben Taheur et al. (2017)
Yeast	*Kloeckera apiculata* 3187, 3188, 3189, 3197, 3198, 3200	University of Sassari (Italy)	0.006	20d	25-40	OTα	NR	liquid laboratory medium	Angioni et al. (2007)
Yeast	*Saccharomyces cerevisiae* 1182, 1236	University of Sassari (Italy)	0.006	20d	34-44	OTα	NR	liquid laboratory medium	Angioni et al. (2007)
Yeast	*Trichosporon sp* DSM 14153, DSM 14156, DSM 14162; *Rhodotorula sp* DSM 14155	DSMZ (Germany)	0.2	5h	80-100	OTα	NR	liquid laboratory medium	Schatzmayr et al. (2003)
Yeast	*Trichosporon mycotoxinivorans* (MTV, 115)	DSMZ (Germany)	0.2	2.5h	100	OTα	NR	liquid laboratory medium	Schatzmayr et al. (2006)
Yeast	*Trichosporon* 178, *Rhodotorula* 124	DSMZ (Germany)	0.2	24h	100	OTα	NR	liquid laboratory medium	Schatzmayr et al. (2006)
Yeast	*Cryptococcus* 118	DSMZ (Germany)	0.2	48h	90	OTα	NR	liquid laboratory medium	Schatzmayr et al. (2006)
Yeast	*Yarrowia lipolytica*	vineyard	1	2d	88	LT, NR	NR	liquid laboratory medium	Yang et al. (2016)

Microorganism type	Species/strain	Source	OTA concentration(μg/mL)	Incubation time	Degradation rate (%)	Degradation products	Degradation enzyme	Reaction conditions	Reference
Yeast	*Phaffia rhodozyma* CBS 5905	NR	7.5	15d	90	OTα	carboxypeptidase(possible)	liquid laboratory medium	Péteri et al. (2007)
Yeast	*Metschnikowia pulcherrima* MACH1, M320; *Kloeckera lindneri* GAL5; *Pichia guilliermondii* M8, M29; *Rhodococcus erythropolis* AR14	Agroinnova culture collection centre (Italy)	7.5	15d	25.8-84	unknown	NR	liquid laboratory medium	Patharajan et al. (2011)
Yeast	*Yarrowia lipolytica* Y-2	Vineyard soil	1	2d	84	NR	NR	liquid laboratory medium	Wang et al. (2014)
Yeast	*Trichosporon mycotoxinivorans* sp. nov.	Mastotermitidae	0.4	2.5h	100	OTα	NR	liquid laboratory medium	Molnar et al. (2004)

NR: Not reported; LT: Some degradation products were notably less toxic than OTA; PBS: Phosphate-buffered saline ; [a]: Cell-free supernatant; [b]: Anaerobic condition ; [c]: μg/g; [d]: The negative control showed 26% OTA loss; [e]: Patent.
