# Supplementary material for: Advances in Biodetoxification of Ochratoxin A-A Review of the Past Five Decades
Source: Front Microbiol. 2018 Jun 26;9:1386. doi: 10.3389/fmicb.2018.01386 (PMC6028724; doi:10.3389/fmicb.2018.01386)
Supplement: Supplementary file 2 [file Table_2.pdf]

Table S2 Summary of OTA adsorbing microorganisms

Microorganism type	Status	Species/strain	Source	OTA concentration(µg/mL)	Incubation time	Degradation rate (%)	Reaction conditions	Reference
Actinobacteria	viable	*Streptomyces* AT10, AT8, SN7, G10, PT1	soil	0.045	1h	16.07-33.93	liquid laboratory medium	El Khoury et al. (2017)
Bacteria	viable	*Bacillus licheniformis* Sl-1	animal faeces	6	24h	60	liquid laboratory medium	Shi et al. (2013)
Bacteria	dead	*Bacillus licheniformis* Sl-1	animal faeces	6	24h	80	liquid laboratory medium	Shi et al. (2013)
Bacteria	viable	*Acetobacter syzygii* KFGM1	Kefir grains	1	24h	15	liquid laboratory medium	Ben Taheur et al. (2017)
Bacteria	viable	*Lactobacillus kefiri* KFLM3	milk	1	24h	15	liquid laboratory medium	Ben Taheur et al. (2017)
Bacteria	viable	*Acetobacter syzygii* KFGM1	Kefir grains	1	24h	50	milk	Ben Taheur et al. (2017)
Bacteria	viable	*Lactobacillus kefiri* KFLM3	milk	1	24h	81	milk	Ben Taheur et al. (2017)
Bacteria	viable	*Oenococcus oeni* RM8, RM11, RM21; *Lactobacillus plantarum* RM28, RM35; *Lactobacillus brevis* RM273; *Leuconostoc mesenteroides* RM54; *Pediococcus acidilactici* RM86	grape must or wine	0.005	48h	8.23-21.10	liquid laboratory medium	Del Prete et al. (2007)
Bacteria	viable	*Lactobacillus brevis* CECT 4669, CECT 4121; *Lactobacillus plantarum* CECT 748	Spanish Type Culture Collection	0.005	48h	8.66-13.42	liquid laboratory medium	Del Prete et al. (2007)
Bacteria	viable	*Oenococcus oeni* Uvaferm ALPHA, MLD; *O. oeni* Viniflora OENOS, CH35	commercial malolactic starter	0.005	48h	17.35-28.09	liquid laboratory medium	Del Prete et al. (2007)
Bacteria	viable	*Bacillus subtilis* CW 14	fresh elk droppings	6	24h	66.6	PBS buffer	Shi et al. (2014)
Bacteria	dead	*Bacillus subtilis* CW 14	fresh elk droppings	6	24h	87.9	PBS buffer	Shi et al. (2014)
Bacteria	viable	*Oenococcus oeni* N171, N172, 6G, 124 M, 228AM, 255 M, S1, eb927/2, MV1, 4100 CECT	wine	0.002	14d	50-63	liquid laboratory medium	Mateo et al. (2010)
Bacteria	viable	*Oenococcus oeni* 6G	wine	0.002	30min	45	liquid laboratory medium	Mateo et al. (2010)
Bacteria	dead	*Oenococcus oeni* 6G	wine	0.002	30min	51	liquid laboratory medium	Mateo et al. (2010)
Bacteria	viable/dead	*Oenococcus oeni* 124 M	wine	0.002	3h	58	liquid laboratory medium	Mateo et al. (2010)
Bacteria	viable	*Lactobacillus plantarum* LOCK 0862, *L. brevis* LOCK 0845, *L. sanfranciscensis* LOCK 0866	Lodz University of Technology	1	24h	14.64-35.01	liquid laboratory medium	Piotrowska (2014)
Bacteria	viable	*Lactobacillus plantarum* LOCK 0862, *L. brevis* LOCK 0845, *L. sanfranciscensis* LOCK 0866	Lodz University of Technology	1	24h	14.80-26.42	PBS buffer	Piotrowska (2014)
Bacteria	dead	*Lactobacillus plantarum* LOCK 0862, *L. brevis* LOCK 0845, *L. sanfranciscensis* LOCK 0866	Lodz University of Technology	1	30min	46.29-59.82	PBS buffer	Piotrowska (2014)

**Microorganism type**	**Status**	**Species/strain**	**Source**	**OTA concentration(µg/mL)**	**Incubation time**	**Degradation rate (%)**	**Reaction conditions**	**Reference**
Bacteria	viable	*Bifidobacterium bifidum* CECT 870T, *B. breve* CECT 4839T*; Lactobacillus casei* CECT 475T, *Lactobacillus casei* CECT 4040, *L. casei* CECT 4045, *L. delbrueckii bulgaricus* CECT 4005, *L. johnsonii* CECT 289, *L. paracasei* CECT 4022, *L. plantarum* CECT 220, *L. plantarum* CECT 221, *L. plantarum* CECT 222, *L. plantarum* CECT 223, *L. plantarum* CECT 748, *L. plantarum* CECT 749, *L. rhamnosus* CECT 278T, *L. rhamnosus* CECT 288, *L. salivarius* CECT 4062[b]	Spanish Type Culture Collection	0.6	24h	1.1-16.1	liquid laboratory medium	Luz et al. (2018)
Filamentous fungi	viable	*Aspergillus niger* GX312, *A. carbonarius* SA332 (a weak OTA producer), *A. Japonicus* AX35	French grapes	2	2h	30, 55, 30	grape juice	Bejaoui et al. (2005)
Filamentous fungi	dead	*Aspergillus niger* GX312, *A. carbonarius* SA332 (a weak OTA producer), *A. Japonicus* AX35	French grapes	2	2h	47.5, 66.5, 41.5	grape juice	Bejaoui et al. (2005)
Filamentous fungi	viable/dead	*Aspergillus niger* GX312, *A. carbonarius* SA332 (a weak OTA producer), *A. Japonicus* AX35	French grapes	0.01	2h	80	grape juice	Bejaoui et al. (2005)
Yeast	immobilisation	*Candida intermedia* 253	University of Sassari	0.02[a]	48h	>80	commercial grape juice	Farbo et al. (2016)
Yeast	viable	*Candida friedrichii* 778, *Candida intermedia* 235, *Lachancea thermotolerans* 751, *Cyberlindnera jadinii* 273	University of Sassari	0.02	8d	70, 73, 75, 0	commercial grape juice	Fiori et al. (2014)
Yeast	dead	*Candida friedrichii* 778, *Candida intermedia* 235, *Lachancea thermotolerans* 751, *Cyberlindnera jadinii* 273	University of Sassari	0.02	8d	72, 74, 84, 82	commercial grape juice	Fiori et al. (2014)
Yeast	viable	*Kazachstania servazzii* KFGY7	Kefir grains	1	24h	6	liquid laboratory medium	Ben Taheur et al. (2017)
Yeast	viable	*Kazachstania servazzii* KFGY7	Kefir grains	1	24h	62	milk	Ben Taheur et al. (2017)
Yeast	viable	*Saccharomyces cerevisiae* RC012, RC016	pig gut	100	1h	71.2-76.6, 71.3-75.9	simulated gastrointestinal conditions	Armando et al. (2012)
Yeast	viable	*Saccharomyces cerevisiae* RC008, 009	feedstuff	100	1h	56.7-82.3, 67.1-80.2	simulated gastrointestinal conditions	Armando et al. (2012)
Yeast	viable	*Saccharomyces cerevisiae* BS	Collection of Industrial Microorganisms of the Institute of Fermentation Technology and Microbiology, Technical University of Lodz (Poland)	1	24h	75	PBS buffer	Piotrowska (2012)
Yeast	dead	*Saccharomyces cerevisiae* BS	Collection of Industrial Microorganisms of the Institute of Fermentation Technology and Microbiology, Technical University of Lodz (Poland)	1	24h	77	PBS buffer	Piotrowska (2012)

Microorganism type	Status	Species/strain	Source	OTA concentration(μg/mL)	Incubation time	Degradation rate (%)	Reaction conditions	Reference
Yeast	viable	*Saccharomyces cerevisiae* S10c, *S. bayanus* S1b, *S. cerevisiae x bayanus* S6u, *Saccharomycodes ludwigii* Slud-1, *Schizosaccharomyces pombe* Schp.3, *Torulaspora delbrueckii* IMIAT-70, *Kloeckera apiculata* IMIAT-111, *Candida pulcherima* IMIAT-179	Italy	0.002	84h	46.83-52.16	white wine must	Cecchini et al. (2006)
Yeast	viable	*Saccharomyces cerevisiae* S10c, *S. bayanus* S1b, *S. cerevisiae x bayanus* S6u, *Saccharomycodes ludwigii* Slud-1, *Schizosaccharomyces pombe* Schp.3, *Torulaspora delbrueckii* IMIAT-70, *Kloeckera apiculata* IMIAT-111, *Candida pulcherima* IMIAT-179	Italy	0.002	84h	53.21-70.13	red wine must	Cecchini et al. (2006)
Yeast	viable/dead	*Debaryomyces hansenii* CYC 1244	NR	7	5min	>98	PBS buffer	Gil-Serna et al. (2011)
Yeast	viable	*Saccharomyces cerevisiae*	AEB Biochemical Inc.	4	90d	73-90	wine must	Csutorás et al. (2013)
Yeast	viable	*Saccharomyces cerevisiae* TP5, TT173	wine	0.004	30d	22.44-81.95	wine	Caridi et al. (2012)
Yeast	viable	*Saccharomyces bayanus* LALVIN QA23	LALLEMAND S.A. company (Montreal, Canada)	0.01	6d	11	red grape juice	Bejaoui et al. (2004)
Yeast	viable	*Saccharomyces cerevisiae* LALVIN BM45, LALVIN Rhône 2056, LALVIN Rhône 2323, LALVIN Rhône 2226, UVAFERM 43	LALLEMAND S.A. company (Montreal, Canada)	0.01	6d	34-45	red grape juice	Bejaoui et al. (2004)
Yeast	dead	*Saccharomyces cerevisiae* LALVIN Rhône 2056	LALLEMAND S.A. company (Montreal, Canada)	2	2h	75	liquid laboratory medium	Bejaoui et al. (2004)
Yeast	viable	*Saccharomyces cerevisiae* LALVIN Rhône 2056	LALLEMAND S.A. company (Montreal, Canada)	2	2h	17	liquid laboratory medium	Bejaoui et al. (2004)
Yeast	dead	*Phaffia rhodozyma* CBS 5905	NR	3	2h	45	liquid laboratory medium	Péteri et al. (2007)
Yeast	viable	*Phaffia rhodozyma* CBS 5905	NR	3	2h	23	liquid laboratory medium	Péteri et al. (2007)
Yeast	viable	*Saccharomyces cerevisiae* Syrena LOCK 0201, Malaga LOCK 0173	Collection of Industrial Microorganisms of the Institute of Fermentation Technology and Microbiology, Technical University of Lodz (Poland)	1	10d	85.1, 82.8	white grape juice	Piotrowska et al. (2013)
Yeast	viable	*Saccharomyces cerevisiae* Syrena LOCK 0201, Malaga LOCK 0173	Collection of Industrial Microorganisms of the Institute of Fermentation Technology and Microbiology, Technical University of Lodz (Poland)	1	10d	65.2, 10.7	blackcurrant juice	Piotrowska et al. (2013)

Microorganism type	Status	Species/strain	Source	OTA concentration(μg/mL)	Incubation time	Degradation rate (%)	Reaction conditions	Reference
Yeast	viable	*Saccharomyces cerevisiae* Syrena LOCK 0201, Malaga LOCK 0173	Collection of Industrial Microorganisms of the Institute of Fermentation Technology and Microbiology, Technical University of Lodz (Poland)	1	24h	21.0, 35.4	liquid laboratory medium	Piotrowska et al. (2013)
Yeast	dead	bakery yeast BS	Collection of Industrial Microorganisms of the Institute of Fermentation Technology and Microbiology, Technical University of Lodz (Poland)	1	24h	64.4	white grape juice	Piotrowska et al. (2013)
Yeast	dead	bakery yeast BS	Collection of Industrial Microorganisms of the Institute of Fermentation Technology and Microbiology, Technical University of Lodz (Poland)	1	24h	62.4	blackcurrant juice	Piotrowska et al. (2013)
Yeast	dead	bakery yeast BS	Collection of Industrial Microorganisms of the Institute of Fermentation Technology and Microbiology, Technical University of Lodz (Poland)	1	24h	54.1	liquid laboratory medium	Piotrowska et al. (2013)
Yeast	viable	*Candida famata* E1, E6, D1, D7,O3; *Candida guilliermondii* S1; *Candida lusitaniae* B1, D2, D9, D11, E2, KK1, KK4, O1; *Cryptococcus laurentii* B4; Kloeckera spp. E3, E4, B2, B3, KK2; *Rhodotorula glutinis* D6	Turkish wine-grapes	0.01	4h	1.96-26.11	PBS buffer	Var et al. (2009)
Yeast	viable	*Candida famata* E1, E6, D1, D7,O3; *Candida guilliermondii* S1; *Candida lusitaniae* B1, D2, D9, D11, E2, KK1, KK4, O1; *Cryptococcus laurentii* B4; Kloeckera spp. E3, E4, B2, B3, KK2; *Rhodotorula glutinis* D6	Turkish wine-grapes	0.01	4h	4.75-21.4	white wine	Var et al. (2009)
Yeast	dead	*Candida famata* E1, E6, D1, D7,O3; *Candida guilliermondii* S1; *Candida lusitaniae* B1, D2, D9, D11, E2, KK1, KK4, O1; *Cryptococcus laurentii* B4; Kloeckera spp. E3, E4, B2, B3, KK2; *Rhodotorula glutinis* D6	Turkish wine-grapes	0.01	4h	4.1-31.31	PBS buffer	Var et al. (2009)
Yeast	dead	*Candida famata* E1, E6, D1, D7,O3; *Candida guilliermondii* S1; *Candida lusitaniae* B1, D2, D9, D11, E2, KK1, KK4, O1; *Cryptococcus laurentii* B4; Kloeckera spp. E3, E4, B2, B3, KK2; *Rhodotorula glutinis* D6	Turkish wine-grapes	0.01	4h	8.08-30.45	white wine	Var et al. (2009)
Yeast	viable	*Saccharomyces cerevisiae* 66 and 35	“Uva di Troia” grape	0.002	NR	6.42-8.59	grape must	Leonardo et al. (2017)

Microorganism type	Status	Species/strain	Source	OTA concentration(µg/mL)	Incubation time	Degradation rate (%)	Reaction conditions	Reference
Yeast	viable	*Saccharomyces cerevisiae* W13, W28, W46, W47, Y28	grape	0.002	5-8d	20.34-76.44	grape must	Petruzzi et al. (2015)
Yeast	viable	*Saccharomyces cerevisiae* W13	grape	0.002	3d	42.8-57.21	liquid laboratory medium	Petruzzi et al. (2014b)
Yeast	viable	*Saccharomyces cerevisiae* W28, W46	grape	0.002	3d	70	liquid laboratory medium	Petruzzi et al. (2014a)
Yeast	viable	*Saccharomyces cerevisiae* Y20, W40	grape	0.002	NR	42.8, 41.4	liquid laboratory medium	Petruzzi et al. (2014c)
Yeast	viable	*Saccharomyces cerevisiae* W13, W47, Y28	grape	0.002	9d	6.70-81.87	synthetic winelike medium	Petruzzi et al. (2014d)
Yeast	viable	*Saccharomyces cerevisiae* RC212, BM45	Lallemand, Montreal, Canada	0.002	9d	3.69-58.55	synthetic winelike medium	Petruzzi et al. (2014d)
Yeast	viable	*Saccharomyces cerevisiae* Y28, W47	grape	0.002	4d	36.96, 42.01	liquid laboratory medium	Petruzzi et al. (2013)
Yeast	viable	*Saccharomyces cerevisiae* 169	collection of the Istituto Sperimentale per l'Enologia	0.004	80d	58.7	red wine	Moruno et al. (2005)
Yeast	viable	*Saccharomyces cerevisiae* 169	collection of the Istituto Sperimentale per l'Enologia	0.007	80d	71.4	red wine	Moruno et al. (2005)
Yeast	viable	*Saccharomyces cerevisiae* EC1118	Lallemand Bio S.L. (Spain)	0.01	2h	28.7	model wine	Nunez et al. (2008)
Yeast	dead	*Saccharomyces cerevisiae* EC1118	Lallemand Bio S.L. (Spain)	0.01	2h	94.9	model wine	Nunez et al. (2008)
Yeast	viable	*Saccharomyces cerevisiae* Lalvin EC1118	Lallemand (Canada)	0.005	7d	25.4	liquid laboratory medium	Bizaj et al. (2016)
Yeast	viable	*Saccharomyces cerevisiae* ZIM 1927	grape must	0.003	7d	29.3	liquid laboratory medium	Bizaj et al. (2016)
Yeast	viable	*Saccharomyces cerevisiae* Lalvin EC1118	Lallemand (Canada)	0.005	7d	13.4-22.0	PBS buffer	Bizaj et al. (2016)
Yeast	viable	*Saccharomyces cerevisiae* ZIM 1927	grape must	0.005	7d	21-38	PBS buffer	Bizaj et al. (2016)
Yeast	dead	*Saccharomyces cerevisiae* Lalvin EC1118	Lallemand (Canada)	0.005	7d	18.2-27.2	PBS buffer	Bizaj et al. (2016)
Yeast	dead	*Saccharomyces cerevisiae* ZIM 1927	grape must	0.005	7d	21.4-54.4	PBS buffer	Bizaj et al. (2016)

NR: Not reported; PBS: Phosphate-buffered saline ; [a]: µg/g; [b]: Anaerobic condition.
