# Supplementary material for: Advances in Biodetoxification of Ochratoxin A-A Review of the Past Five Decades
Source: Front Microbiol. 2018 Jun 26;9:1386. doi: 10.3389/fmicb.2018.01386 (PMC6028724; doi:10.3389/fmicb.2018.01386)
Supplement: Supplementary file 3 [file Table_3.pdf]

Table S3 Summary of OTA degrading enzymes

Producer	Status	Source	Enzyme name	OTA concentration(μg/mL)	Incubation time	Degradation rate (%)	Degradation products	Reaction conditions	pH	Temperature (ºC)	Reference
laboratory	crude	*Aspergillus tubingensis* M036	NR	0.04	24h	97.5	OTα	phosphate-citrate buffer	5	25	Cho et al. (2016)
laboratory	crude	*Aspergillus tubingensis* M074	NR	0.04	24h	91.3	OTα	phosphate-citrate buffer	5	25	Cho et al. (2016)
laboratory	crude	*Bacillus amyloliquefaciens* ASAG1	carboxypeptidase	10	overnight	41	OTα	Tris-HCl	7	NR	Chang et al. (2015)
laboratory	purified	*Bacillus amyloliquefaciens* ASAG1	carboxypeptidase	10	overnight	72	OTα	Tris-HCl	7	NR	Chang et al. (2015)
laboratory	crude	*Aspergillus niger* MUM 03.58	Ancex	1	25h	99.8	OTα	phosphate buffer	7.5	37	Abrunhosa et al. (2006)
laboratory	crude	*Aspergillus niger* MUM 03.58	Ancex	1	3h	87.9	OTα	phosphate buffer	7.5	50	Abrunhosa et al. (2006)
commercial corporation	purified	*Aspergillus niger*	Protease A	1	25h	87.3	OTα	phosphate buffer	7.5	37	Abrunhosa et al. (2006)
commercial corporation	purified	Porcine pancreas	Pancreatin	1	25h	43.4	OTα	phosphate buffer	7.5	37	Abrunhosa et al. (2006)
commercial corporation	purified	*Aspergillus niger*	Prolyve PAC	1	25h	3	OTα	citrate buffer	3	37	Abrunhosa et al. (2006)
commercial corporation	purified	bovine pancreas	carboxypeptidase A	1	9h	100	OTα	Tris buffer	8.5	37	Abrunhosa et al. (2006)
laboratory	crude	*Aspergillus niger*	metalloenzyme	1	24h	>95	OTα	phosphate buffer	7.5	37	Abrunhosa and Venâncio (2007)
laboratory	crude	*Phaffia rhodozyma* CBS 5905	carboxypeptidase	5	72h	66	OTα	liquid laboratory medium	NR	30	Péteri et al. (2007)
laboratory	crude	*Aspergillus niger* MUM 03.58	hydrolase	1000	4h	NR	OTα	phosphate buffer	7.5	37	Abrunhosa et al. (2011)
laboratory	purified	*Aspergillus niger*	ochratoxinase	0.05	1h	50	OTα	Mops/HCl	7.5	40	Dobritzsch et al. (2014)
commercial corporation	purified	bovine pancreas	carboxypeptidase A	0.07	18d	100	OTα	liquid laboratory medium	7.5	25	Deberghes et al. (1995)
laboratory	purified	*Aspergillus niger*	amidase 2	0.85	30min	83	OTα	Mops-NaOH	7	40	Yu et al. (2015)[b]
laboratory	purified	*Aspergillus niger*	amidase 2	0.047	2.5h	>95.7	OTα	milk	NR	40	Yu et al. (2015)[b]
laboratory	purified	*Aspergillus niger*	amidase 2	38	20h	>99.9	OTα	corn flour-Mops-NaOH	7	30	Yu et al. (2015)[b]
laboratory	purified	*Aspergillus niger*	amidase 2	38	20h	82	OTα	corn soy based feed-fermentation broth	NR	NR	Yu et al. (2015)[b]
commercial corporation	purified	*Saccharomyces cerevisiae*	carboxypeptidase Y	1	5d	52	OTα	NR	5.6	37	Abrunhosa et al. (2010)
commercial corporation	purified[a]	*Aspergillus niger*	lipase A	50	2h	100	OTα	sodium phosphate buffer	7.5	37	Stander et al. (2000)
laboratory	crude	*Acinetobacter* sp. *neg1*	carboxypeptidase PJ_1540	1	overnight	33	OTα	Tris buffer	NR	28	Liuzzi et al. (2016)
laboratory	crude	*Stenotrophomonas* sp. CW117	NR	0.02	72h	83.1	NR	NR	NR	NR	Jiang et al. (2016b)[b]
laboratory	crude	*Luteimonas* sp. CW574	NR	0.02	48h	17.7	NR	NR	NR	NR	Jiang et al. (2016d)[b]
laboratory	crude	*Silanimonas* sp. CW282	NR	0.02	48h	38.9	NR	NR	NR	NR	Jiang et al. (2016c)[b]
laboratory	crude	*Lysobacter* sp. CW239	NR	0.02	48h	77.1	NR	NR	NR	NR	Jiang et al. (2016a)[b]

NR: Not reported; PBS: Phosphate-buffered saline ; [a]: It was purified from crude lipase (purchased from Amano) by authors themselves; [b]: Patent.
